# Enhancing paranasal sinus disease detection with AutoML: efficient AI development and evaluation via magnetic resonance imaging

**DOI:** 10.1007/s00405-023-08424-9

**Published:** 2024-01-10

**Authors:** Ryan Chin Taw Cheong, Susan Jawad, Ashok Adams, Thomas Campion, Zhe Hong Lim, Nikolaos Papachristou, Samit Unadkat, Premjit Randhawa, Jonathan Joseph, Peter Andrews, Paul Taylor, Holger Kunz

**Affiliations:** 1 Royal National ENT and Eastman Dental Hospitals, University College London Hospitals NHS, London, UK; 2 Barts Health NHS Trust, London, UK; 3 University College London, London, UK; 4 Medical Physics and Digital Innovation Laboratory, School of Medicine, Aristotle University of Thessaloniki, Thessaloniki, Greece; 5 School of Public Health, Imperial College London, London, UK

**Keywords:** AutoML, Automated machine learning, Paranasal sinus disease, MRI, Artificial intelligence

## Abstract

**Purpose:**

Artificial intelligence (AI) in the form of automated machine learning (AutoML) offers a new potential breakthrough to overcome the barrier of entry for non-technically trained physicians. A Clinical Decision Support System (CDSS) for screening purposes using AutoML could be beneficial to ease the clinical burden in the radiological workflow for paranasal sinus diseases.

**Methods:**

The main target of this work was the usage of automated evaluation of model performance and the feasibility of the Vertex AI image classification model on the Google Cloud AutoML platform to be trained to automatically classify the presence or absence of sinonasal disease. The dataset is a consensus labelled Open Access Series of Imaging Studies (OASIS-3) MRI head dataset by three specialised head and neck consultant radiologists. A total of 1313 unique non-TSE T2w MRI head sessions were used from the OASIS-3 repository.

**Results:**

The best-performing image classification model achieved a precision of 0.928. Demonstrating the feasibility and high performance of the Vertex AI image classification model to automatically detect the presence or absence of sinonasal disease on MRI.

**Conclusion:**

AutoML allows for potential deployment to optimise diagnostic radiology workflows and lay the foundation for further AI research in radiology and otolaryngology. The usage of AutoML could serve as a formal requirement for a feasibility study.

## Introduction

Automated machine learning (AutoML) aims to make high-quality machine learning accessible directly to knowledge domain experts (such as physicians) who lack the resources to implement a machine learning algorithm [1]. AutoML encapsulates the stages of a machine learning and data analytics pipeline after data collection (data cleaning, feature engineering, model discovery, model selection, hyperparameter optimisation, model performance evaluation, and model deployment) into a “sealed box” [1].

Only 1% of UK trusts and health boards achieved their radiology reporting targets, with a growing backlog of 12,000 cross-sectional studies and 200,000 plain radiographs since 2016 [2]. One way to ameliorate these pressures would be to automate elements of the radiologists’ work.

Convolutional Neural networks (CNNs) are a specialised kind of neural network for processing data that has a grid-like topology [3]. A CNN consists of several layers: convolutional, pooling, and fully connected layers [3]. Each convolutional layer consists of a certain number of trainable parametric filters [3]. Each convolutional layer is typically followed by a pooling layer which reduces the feature space [3]. Finally, the data are passed to one or more fully connected layers and the predicted output is produced [3]. CNNs trained on CT-paranasal sinus data have shown good diagnostic accuracy in differentiating anterior ethmoidal artery position at the skull base or hanging on a bony mesentery, which has significant clinical implications in surgical planning, in minimising complications and in optimising patient outcomes [4]. A CNN has also been trained to detect pneumatisation of the middle turbinate on CT-paranasal sinuses with high accuracy, which supports further research in clinically relevant anatomical variations in otolaryngology [5]. A CNN for automated classification of paranasal sinus opacification on CT in a population with a range of sinonasal inflammations has automatically segmented the paranasal sinuses to produce scores that are concordant with Lund-MacKay visual scoring, and showed that CNN-based opacification scores correlate with asthma diagnoses and chronic rhinosinusitis [6].

The rationale for this study is to show that Vertex AI [7], the AutoML platform recently launched by Google Cloud, is able to classify the absence or presence of paranasal sinus disease on the images from the Open Access Series of Imaging Studies (OASIS-3) [8] MRI radiological dataset. AutoML can make technical science of AI “clinician-ready” and support the development of a potential Clinical Decision Support System (CDSS) for screening purposes.

## Methods

### Setting

The Open Access Series of Imaging Studies (OASIS)-3 is a retrospective compilation of anonymized neuroimaging data for more than 1000 participants that were gathered through the Washington University in St Louis (WUSTL) Knight Alzheimer's Disease Research Centre (ADRC) over 30 years [8]. Participants include 609 cognitively normal adults and 489 individuals at various stages of cognitive decline ranging from 42 to 95 years of age. All participants were assigned a new random identifier, and all dates were removed and normalised to reflect days from entry into study. The dataset contains 2168 MRI sessions and 1608 positron emission tomography (PET) sessions.

### Dataset

Purposive sampling was used to select 1383 unique T2-weighted (T2w), non-turbo spin echo (TSE) MRI head sessions. T2w MRI were selected as it displays paranasal sinus disease with remarkable clarity [9] and TSE MRI sessions were excluded due to the reduced number of interleaved image slices that can be obtained [10]. The coronal anatomical imaging plane slice number 180 was selected on T2w MRI displaying both the ethmoid and maxillary sinuses bilaterally to enable maximum display of information of the paranasal sinuses on a single two-dimensional (2D) slice of imaging.

Inclusion criteria:

1. T2-weighted, non-TSE MRI head sessions in the coronal anatomical plane in the OASIS-3 dataset displaying both the ethmoid and maxillary sinuses bilaterally.

Exclusion criteria:

1. Images with insufficient visual or radiological information
2. Non-MRI data in the OASIS-3 dataset
3. T1-weighted MRI in the OASIS-3 dataset
4. TSE MRI sessions.

Any deviation from what is considered a radiologically normal paranasal sinus from baseline by our expert consultant radiologists is labelled as ‘disease’ and this includes abnormal mucosal thickening and potential sinonasal malignancy. AutoML in the form of the Vertex AI [7] image classification model was chosen to automate the training, validation, and testing stage of the AI algorithm, hyperparameter tuning, and the model performance evaluation to reduce the barrier of entry for physicians.

### Measurements

The study used a PC with Intel(R) Core (TM) i7-8750H Central Processing Unit (CPU) @ 2.20 GHz processor and 16.0 GB RAM. The percentage of consensus between the three specialised head and neck consultant radiologists and the average time spent labelling is automatically measured by the Labelbox platform [11]. The consensus labelled data set is split into training, validation, and test groups in a ratio of 80:10:10, respectively. K-fold cross-validation is frequently used method for error estimation and testing of the success rate of models used for classification [12]. In this study, k-fold cross-validation with a k value of 10 was selected, as 10 has been shown to be an optimal value for error reduction [12].

### Procedures and data pre-processing

**Fig. 1:**
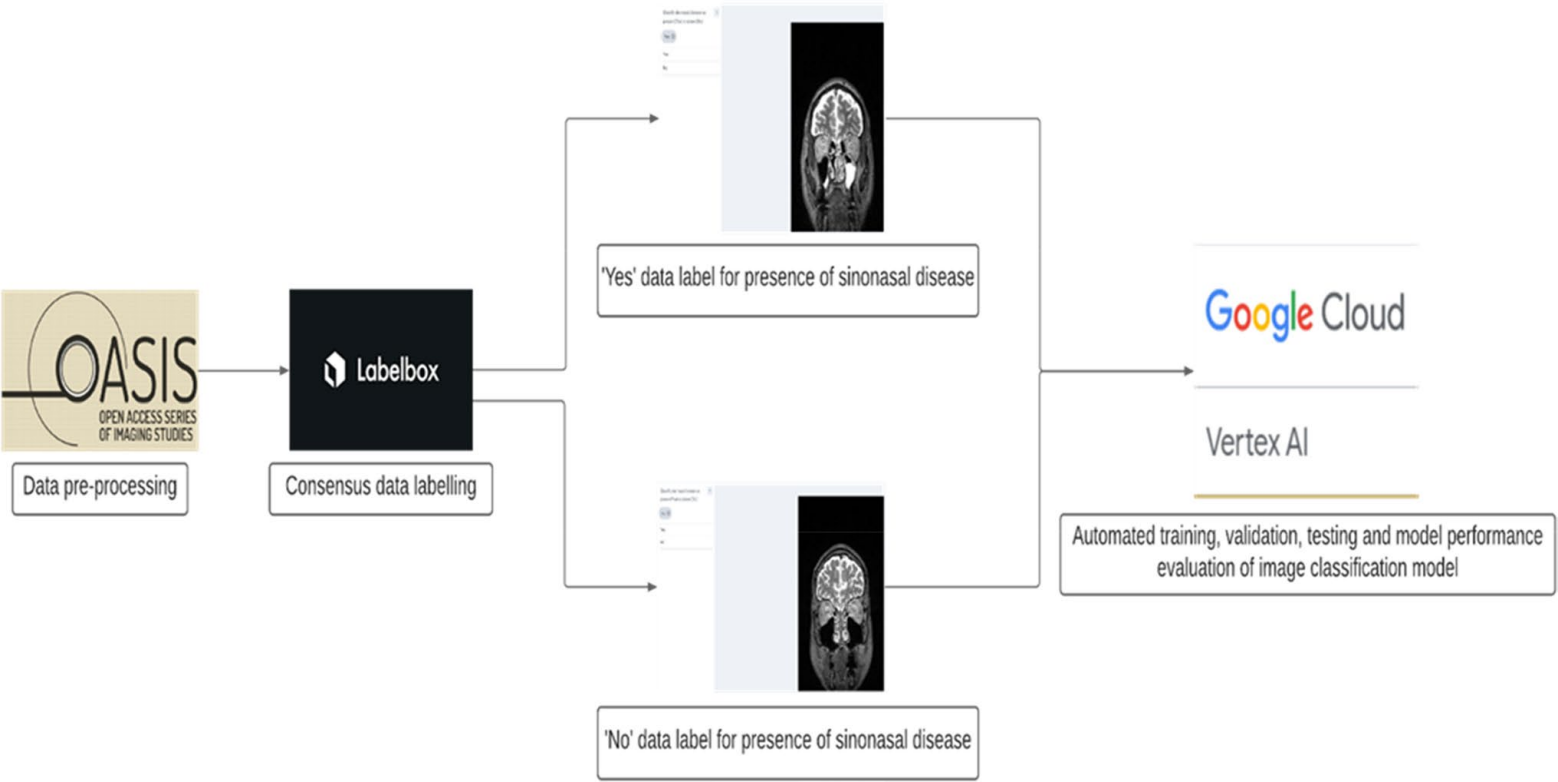
Schematic pipeline of study design

**Fig. 2:**
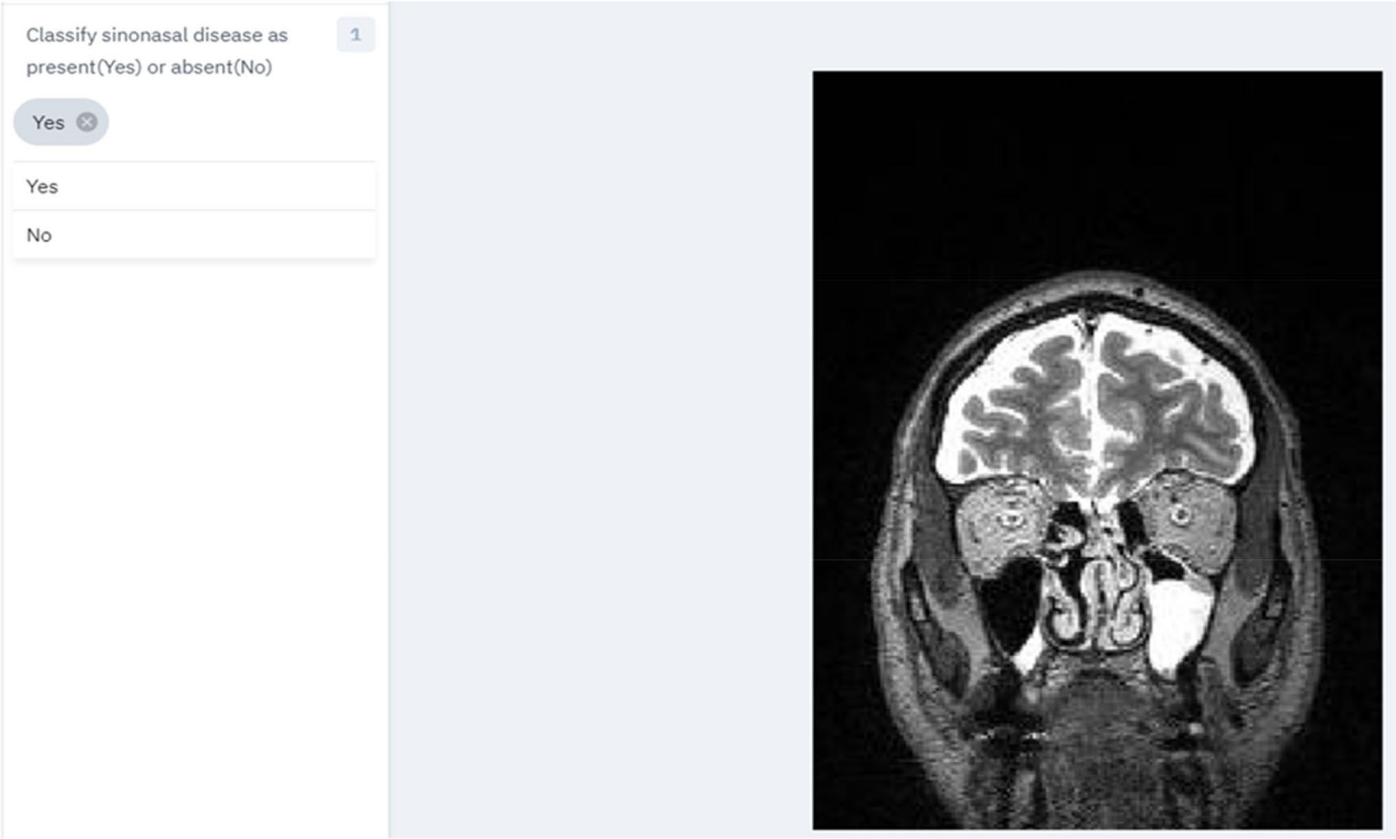
Labelbox dropdown interface ‘Yes’ sinonasal disease label performed by expert clinician data labellers

**Fig. 3:**
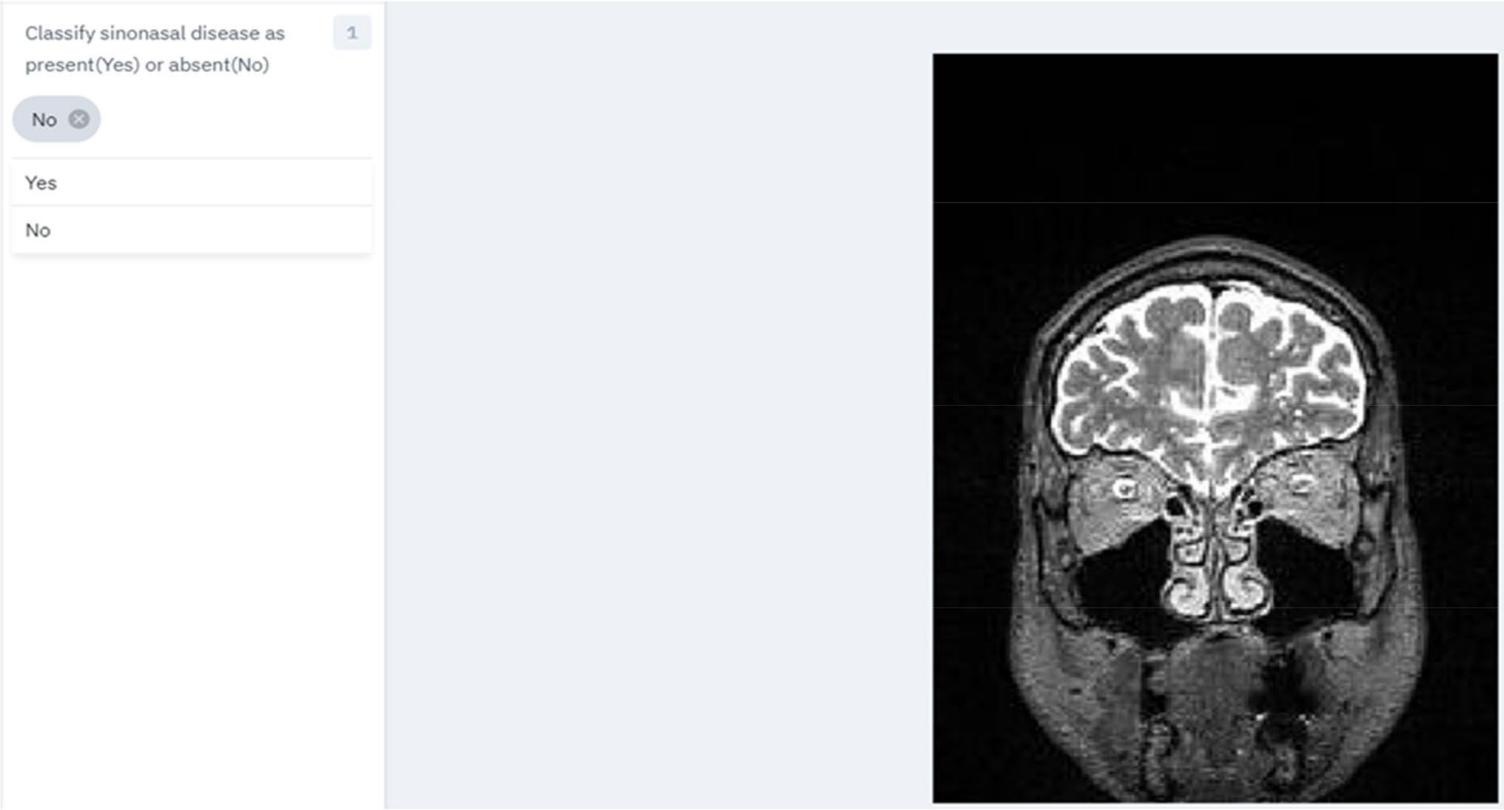
Labelbox dropdown interface ‘No’ sinonasal disease label performed by expert clinician data labellers

The OASIS-3 dataset was accessed after application via the website link https://www.oasis-brains.org/ and subsequently available for download through the XNAT Central publicly accessible medical imaging data repository on https://central.xnat.org/ [13]. Image data in Neuroimaging Informatics Technology Initiative (NIfTI) format and scan type of MRI T2w were selected prior to ZIP download of the dataset. The schematic pipeline of the study design is depicted in Fig. 1. The feature selection of a single image 2D de-identified non-TSE T2w MRI in the coronal plane slice number 180 of pixel dimensions of 176(width) × 256(height) was selected and uploaded to the Labelbox platform. Examples of the Labelbox interface visualised independently by the three specialised head and neck consultant radiologists is shown with dropdown selection of labels ‘Classify sinonasal disease as present (Yes)’ in Fig. 2 and ‘Classify sinonasal disease as present (No)’ in Fig. 3. Images with insufficient visual or radiological information were excluded during the pre-processing of the dataset and the consensus data labelling stage as per the exclusion criteria. Upon completion of the consensus data labelling process, all the labelled data were exported from the Labelbox platform to a comma-separated values (CSV) format. A label of either ‘Yes’ or ‘No’ is assigned when there is a baseline majority consensus of at least 2 out of 3, or full consensus of 3 out of 3 of the independent specialised head and neck consultant radiologists data labellers. The total combined expert clinician labelling time was 4 h and 7 min.

Automated analysis of the consensus data labelling generates the time taken to complete the data labelling process for each of the three head and neck consultant radiologists and the consensus percentage. Vertex AI generates model evaluation measures, such as the accuracy, sensitivity, and precision as a measure of image classification model performance [14]. The definition of basic performance metrics can be obtained from a standard textbook in machine learning (see [15, 16]).

## Results

A total of 1383 unique non-TSE T2w MRI head sessions were downloaded from the OASIS-3 dataset with 2 sessions manually excluded due to insufficient visual or radiological information, leaving 1381 single coronal sliced sessions which were uploaded onto Labelbox. A further 5 images were excluded during the consensus data labelling process due to insufficient visual or radiological information, leaving 1376 consensus labelled data, consisting of 599 ‘No’ sinonasal disease labels and 777 ‘Yes’ sinonasal disease labels to be used in ratio of 80% (1100 labels) training, 10% (138 labels) validation and 10% (138 labels) testing. Labelbox automatically generated the average consensus percentage between the three data labellers at 77%. The average time taken for a label to be assigned by a head and neck consultant radiologists was 5 s. All ten iterations of the k-fold cross-validation consensus labelled datasets each containing 1376 data labels were successfully trained on the Vertex AI image classification (Single-label) model on the Google Cloud Platform interface, as shown in Fig. 1.

The best-performing image classification model achieved a sensitivity of 91.3%, specificity (precision) 92.8%, and accuracy of 92%. The frequency of true positives, true negatives, false positives, and false negatives are: TP 63, TN 64, FN 6, and FP 5. The Vertex AI image classification model trained on the consensus labelled open access OASIS-3 dataset for paranasal sinus disease on MRI had an average training time of 158.5 min. The final trained model is available as an exported file upon request.

## Discussion

In its current state and form, the Vertex AI MRI paranasal sinus disease image classification model could serve as a screening tool and clinical decision support system (CDSS) to streamline radiological workflows by filtering MRI scans detected as having paranasal sinus disease for a human radiologist to review, whilst excluding MRI scans classified as not having paranasal sinus disease. This improvement in efficiency could allow radiologists to focus their expertise on MRI scans that require their attention, thereby reducing their workload [17]. More judicious use of rare and expensive human machine learning expertise [18] will allow knowledge domain experts such as otolaryngologists to focus more on identifying problems of high clinical value that are currently less amenable to AI solutions.

The ‘Deploy Model’ function available within the Google Cloud platform can allow the trained Vertex AI model to be deployed into low- and middle-income countries (LMIC) where access to radiology resources is restricted [19].

A high average consensus rate of 77% between the most specialised and experienced human data labellers has resulted in a robustly labelled, high-quality dataset that could be used as a radiological education tool in regions with limited access to the expertise of specialised head and neck consultant radiologists.

A clinical limitation of the study is that although an appropriately selected single coronal 2D slice of MRI can display significant views of the maxillary and ethmoidal sinuses, it does not encompass all the remaining axial and sagittal anatomical planes and less commonly affected frontal and sphenoid sinuses. A potential technical limitation to generalizability at the deployment stage is the tolerance of variation in data structure and form of the real-world MRI scans presented as inputs to the trained algorithm, which will be different to the machine-readable format of the OASIS-3 dataset. A further clinical limitation is that although potentially valuable in terms of radiological screening and detection of severe paranasal sinus pathology, incidental inflammatory paranasal sinus disease on radiological imaging such as MRI may not correlate strongly with clinical severity of patient symptoms [20].

To ensure a more comprehensive review of the MRI scans, further studies in multi-slice [21] and multi-label [22] model training of the Vertex AI image classification model with more specific radiological data labels of paranasal sinus diseases, such as inverted papillomas, fungal sinusitis, and sinonasal malignancies incorporating the axial and sagittal anatomical planes will be the next consideration. Further work in the form of saliency mapping will also be considered to identify areas of an input MRI scan used by AutoML to make its decisions [23].

Clinically, this study demonstrates the feasibility and high performance of the Vertex AI image classification model to detect the presence or absence of sinonasal disease on MRI, and this conclusion shows promise for potential deployment to optimise diagnostic radiology workflows. In addition, the machine-readable, standardised, de-identified MRI dataset with expert consensus labelled data on the presence or absence of sinonasal disease is of considerable value when shared with the AI and machine learning community.

A key technical feature of the approach used was the saving in time: developing, coding, and implementing a similar ML pipeline in a programming language, such as Python or R, would have required substantial effort and incurred considerable cost. AutoML could thus serve as an agile means of assessing whether a project is fundamentally feasible before a further allocation of resources follows.

The clinical quality of the outputs and the nature of the platform on which they were achieved serve to demonstrate the feasibility of partitioning the healthcare machine learning task, such that it is the clinical domain experts who undertake the primary work with advice from ML experts and not vice versa. This lays a foundation for further domain-expert-led AI research across healthcare.

## Data Availability

The datasets derives from a public repository OASIS-3 MRI radiological dataset [8].
